# Advanced High‐Throughput Root Phenotyping and GWAS Identifies Key Genomic Regions in Cowpea During Vegetative Growth Stage

**DOI:** 10.1111/ppl.70375

**Published:** 2025-07-03

**Authors:** Liny Lay, Sheikh Mansoor, Waleed Khan, Mohammad Shafiqul Islam, Amit Ghimire, Hyun Jo, Yong Suk Chung, Yoonha Kim

**Affiliations:** ^1^ Department of Applied Biosciences Kyungpook National University Daegu Republic of Korea; ^2^ Department of Extension for Agriculture, Forestry and Fisheries MAFF Phnom Penh Cambodia; ^3^ Department of Integrative Biology Kyungpook National University Daegu Republic of Korea; ^4^ Department of Plant Resources and Environment Jeju National University Jeju Republic of Korea; ^5^ Department of Agriculture Noakhali Science and Technology University Noakhali Bangladesh; ^6^ Upland Field Machinery Research Center Kyungpook National University Daegu Republic of Korea

**Keywords:** advance phenotyping, genotyping, root architecture, single nucleotide polymorphisms, *Vigna unguiculata*

## Abstract

Improving crop production in changing environments can be achieved through selective breeding; however, limited advanced root phenotyping and genotyping in early growth stages hinder assessing root architecture variation and diversity, despite its importance. Therefore, this study utilized advanced image phenotyping on a diverse set of 222 cowpea accessions, revealing significant variations in key phenotypic traits and the genomic regions influencing them. Our study revealed a total of 55 genes linked to major root traits. Among eight root traits—total root length (TRL), surface area (SA), average diameter (AD), root volume (RV), tip number (TN), fork number (FN), primary root length (PRL), and lateral root length (LRL), analyzed, seven significant single nucleotide polymorphisms (SNPs) demonstrated particularly strong associations with three key traits, including surface area (SA), tip number (TN), and fork number (FN). SA emerged as a significant trait, exhibiting considerable variation across the studied accessions. The mean SA was 59.59 cm^2^, with some genotypes surpassing 140.72 cm^2^. Further analysis identified two SNPs that showed significant association with SA, located on two distinct chromosomes: 3 and 11. Similarly, two significant SNPs associated with TN were found on chromosome 3, while three SNPs associated with FN were identified on chromosomes 2, 3, and 8. These findings significantly advance our understanding of the genetic foundations underlying important phenotypic traits in cowpeas, offering a robust framework for future genetic improvement initiatives. The results strongly suggest that implementing breeding programs focused on selecting root phenotypes could significantly enhance cowpea productivity across various environments.

## 1 Introduction

Cowpea ( *Vigna unguiculata* L. Walp) is a crucial protein source and food security crop for numerous communities across Africa, Asia, and South America. This legume not only enhances nitrogen levels in agroecosystems but also provides valuable fodder for livestock, particularly in low‐input agricultural systems prevalent in its growing regions (Snapp et al. 2018; Ojiewo et al. 2019). As a nitrogen‐fixing legume, cowpea plays a vital ecological role, with its non‐grain biomass serving as a significant component of livestock diets. Thriving in warm climates, cowpea requires less rainfall than most other crops, including rice, wheat, maize, soybean, and cotton, making it ideally suited for semi‐arid and arid regions of the lowland tropics and subtropics (Dogan et al. 2007; Emongor 2007; Wang et al. 2012; Lamb et al. 2015; Wang 2017). Its adaptability to marginal environments characterized by drought, low soil fertility, and pest pressures—factors likely to intensify due to climate change—further underscores its importance (Yadav et al. 2015). To prosper in resource‐limited conditions, cowpeas must efficiently acquire soil resources, a capacity that can be enhanced through the development of a robust root system architecture. While most breeding programs have focused on improving above‐ground traits, there remains an underexploited strategy of trait‐based selection that links specific root traits to enhanced resource acquisition (Cattivelli et al. 2008; J. P. Lynch 2015). This approach offers promising avenues for improving cowpea's resilience and productivity in challenging environments.

While breeding programs predominantly focus on enhancing crop productivity and resilience, relatively few have explored the potential of selecting specific traits (Wasson et al. 2012; York et al. 2013). A notable exception is found in common bean ( *Phaseolus vulgaris* ) breeding, where trait‐based selection has been successfully implemented by targeting specific root characteristics, such as root hair length and density. This approach has proven highly effective in achieving desired outcomes in crop improvement (Burridge et al. 2019). Traits, the fundamental units of plant phenotype akin to genes in genotype, must be validated for their utility before incorporation into selection criteria (York et al. 2013). This validation process involves developing robust screening methods and quantifying both genetic variation and heritability (Tracy et al. 2020). Significant genetic variation for beneficial root traits has been identified in crops such as maize and soybean through phenotyping at both seedling and mature plant stages (Gao and Lynch 2016; Vandamme et al. 2016; Prince et al. 2019). Root hair length and density are crucial root phenes for efficient uptake of immobile soil resources, particularly phosphorus, and can be accurately assessed during the seedling stage (J. P. Lynch 2013, 2019; Zhang et al. 2018). These root hairs, subcellular projections found in most vascular plants, serve multiple functions: they enhance water and nutrient uptake, facilitate penetration of compacted soil, and substantially increase the root crown's surface area (Paez‐Garcia et al. 2015).

Incorporating root traits into crop breeding presents significant challenges, primarily due to the complexity of phenotyping mature root systems in field conditions (Thomas et al. 2016; Strock et al. 2019). In contrast, phenotyping at the seedling stage offers a simpler and more cost‐effective approach, circumventing the difficulties associated with field‐grown plants while still providing valuable insights into the root architecture (RA) of mature plants (Orman‐Ligeza et al. 2014; Mansoor and Chung 2024). However, visualizing and measuring RA traits for conventional breeding remain problematic. The dynamic nature of root systems and the transient expression of many RA characteristics led to considerable variability even within the same genotype (Orman‐Ligeza et al. 2014). This variability complicates the selection process and underscores the need for robust phenotyping methods. To optimize breeding effectiveness and minimize costs, RA trait measurement should be strategically targeted and tailored to specific environmental conditions (de Dorlodot et al. 2007). This targeted approach allows breeders to focus on the most relevant traits for a given environment, thereby improving the efficiency of selection and the likelihood of developing cultivars with enhanced root systems suited to their intended growing conditions.

Root phenotyping is equally crucial as shoot phenotyping, given that roots play a pivotal role in water and nutrient absorption, directly influencing plant performance (Wasaya et al. 2018). In the context of cowpea improvement, understanding the root system's function during early vegetative growth is of paramount importance. Although research on the genetic and phenotypic variation of cowpea root architecture (RA) traits during this critical stage remains limited, significant genetic diversity has been observed in cowpea for RA traits that enhance growth in nutrient‐poor and arid conditions. This diversity underscores the importance of root traits in adapting cowpea to challenging environments (Burridge et al. 2016, 2017; Gull et al. 2020; Mohammed et al. 2022; Zaffar et al. 2024).

Plants possess the ability to adjust their root architecture in response to environmental stresses through genetic programs that regulate root growth (Jovanovic et al. 2007). Studies on genomic regions controlling root phenotypic plasticity under drought stress in cereal crops indicate that root system plasticity offers valuable genetic variation for stress adaptation (Sandhu et al. 2016; Kadam et al. 2017; Schneider, Klein, Hanlon, Kaeppler, et al. 2020; Schneider, Klein, Hanlon, Nord, et al. 2020; Mansoor, Karunathilake, et al. 2024; Mansoor, Tripathi, et al. 2024). Identifying novel quantitative trait loci (QTLs) is crucial for exploring genetic diversity in root system traits (Schneider and Lynch 2020). Genome‐wide mapping techniques have been employed to discover new genetic loci linked to root architecture across various populations (Siddiqui et al. 2021). Advancements in genome sequencing and bioinformatics have enhanced methods such as association and linkage mapping, improving our understanding of genetic diversity in crops (Kumar et al. 2015; Mansoor, Hamid, et al. 2024). Genome‐wide association studies (GWAS) are frequently used to identify new genes and QTLs associated with traits such as growth, stress tolerance, and nutritional quality. However, there are few reports on mapped genes or single nucleotide polymorphisms (SNPs) associated with root traits in cowpeas at the vegetative stage (Gull et al. 2020; Mohammed et al. 2022).

Root development is a crucial determinant of plant performance; however, genetic mapping of root traits in cowpea has predominantly relied on conventional phenotyping methodologies. Despite these efforts, previous studies have not established a clear link between root phenotypes at the vegetative stage and overall plant performance. This study aims to address this gap by identifying SNPs associated with the regulation of root architecture in cowpea during the vegetative growth stage (V2). Additionally, the study seeks to elucidate the correlations between root traits observed during this critical developmental period and other agronomically important traits by leveraging advanced high‐throughput phenotyping.

## 2 Materials and Methods

### 2.1 Plant Material and Conditions

In this study, 222 landrace accessions from 46 countries spanning six continents were examined (Table S1). The seeds exhibited a germination rate between 70% and 85%. Our experiment was carried out two times in 2023, first from August 17 to September 4, and then from October 12 to November 23 at a greenhouse located at Kyungpook National University in Daegu. Seeds were planted in polyvinyl chloride (PVC) pipes, each 7 cm in diameter and 40 cm in height, filled with sandy soil. When the plants reached the vegetative growth stage (V2), root samples were harvested. During the experiment, plants were regularly irrigated, and the temperature was maintained within an ideal range of 30°C ± 3°C. Each experiment included three replications, resulting in a total of six replications for this study. However, to minimize variation among the accessions, we selected four replications by excluding the outliers from the six replications, thereby enhancing the consistency of the results.

### 2.2 Image Acquisition, Analysis and Trait Measurements

**FIGURE 1:**
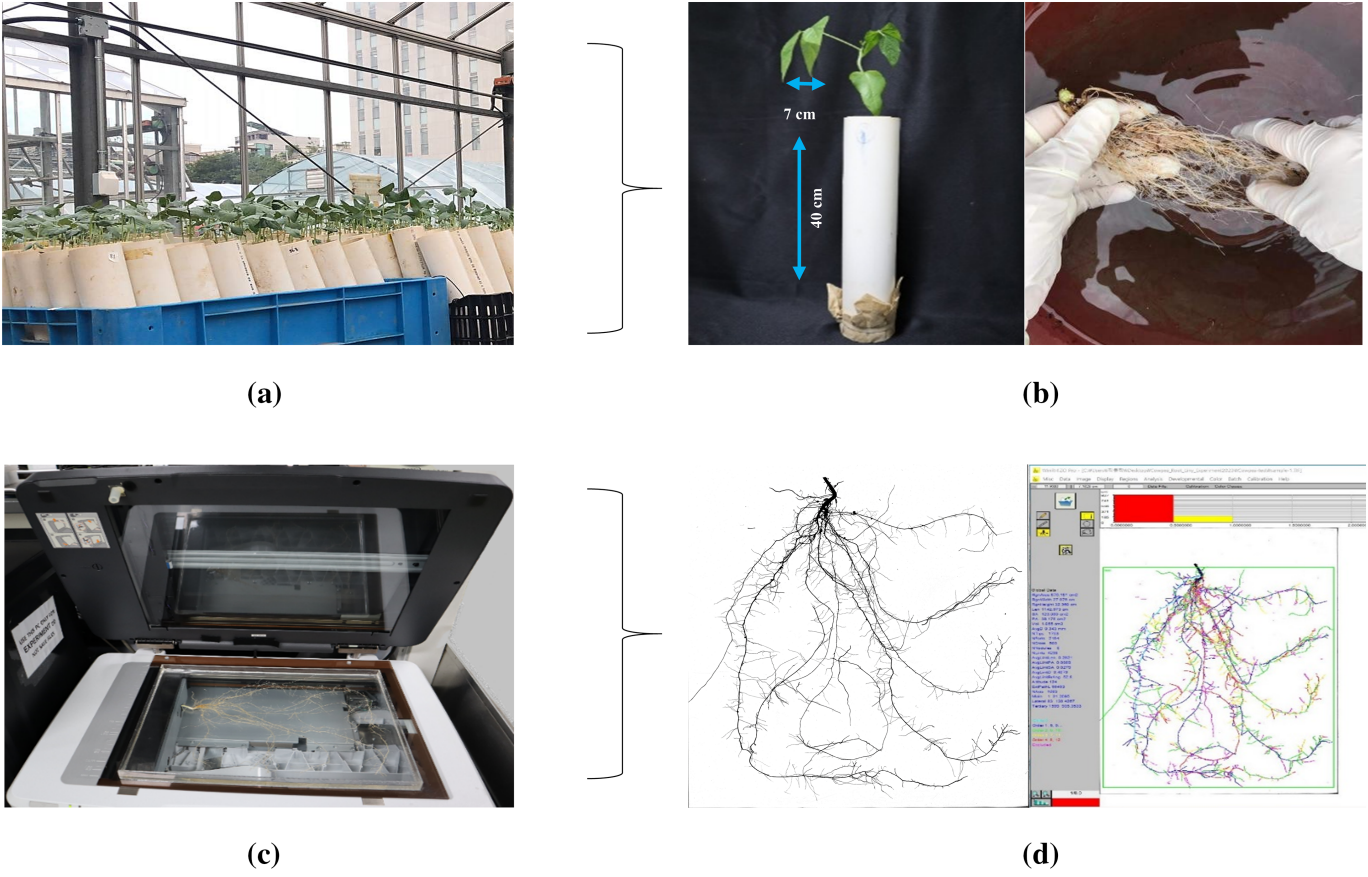
Workflow for root phenotype collection: (a) Seedlings at the V2 growth stage grown in PVC pipes within the greenhouse. (b) Cowpea plants with roots cleaned to remove soil particles. (c) Roots, after cleaned with tap water, were placed in a container and scanned using an EPSON scanner. (d) 2‐D images of the scanned roots were analysed using WinRHIZO Pro software.

Roots from all samples were harvested upon reaching the V2 stage. The roots were carefully extracted from the sandy soil and rinsed with tap water to eliminate any remaining soil particles. To prevent desiccation and preserve root structure, they were then stored in zip‐lock plastic bags filled with water. Subsequently, the roots were scanned with an EPSON scanner (Expression 12000XL manufactured by Epson) to obtain 2‐D images. The root samples were carefully taken out of the zip‐lock plastic bags and placed on a transparent tray (40 × 30 × 2 cm) filled with clean water before scanning. This setup was designed to separate and reduce the overlapping of roots during scanning. These images were analyzed for root morphological traits using WinRHIZO Pro software (Regent Instrument Inc.). Work procedures for data collection are depicted in Figure 1a–d. Among eight analyzed traits—total root length (TRL), surface area (SA), average diameter (AD), root volume (RV), tip number (TN), fork number (FN), primary root length (PRL), and lateral root length (LRL). These three traits are essential for plant growth and development. SA presents the total area of all roots, a key parameter in root morphology that significantly impacts a plant's ability to absorb water and nutrients from the soil (Hodge et al. 2009; Zobel and Waisel 2010; Tripathi et al. 2021). Similarly, TN is the total count of root tips within a plant's root system, which are the primary growth points of roots crucial for penetration, exploration, and nutrient uptake (J. Lynch 1995; Gorim and Vandenberg 2017). Additionally, FN is the number of branching points where roots divide into two or more and serves as a critical indicator of the root system's complexity and its potential to explore soil volume (Fitter and Stickland 1991).

### 2.3 Genome‐Wide Association Analysis

The GWAS analysis was performed using the R‐package GAPIT3 (Genome Association and Prediction Integrated Tool) (Bradbury et al. 2007; Lipka et al. 2012). Genotyping of the 280 cowpea accessions was carried out with the Illumina Cowpea iSelect Consortium Array, which includes 51,128 SNPs. The initial dataset was filtered to ensure quality by removing SNPs with a minor allele frequency (MAF) of less than 5%, excessive missing data, or heterozygous calls in more than 10% of the accessions. Additionally, filtering was performed using TASSEL 5.2.9, resulting in the exclusion of cowpea accessions with more than 20% missing SNPs or heterozygous calls. After these steps, 39,056 high‐quality SNPs were retained for analysis. GWAS was then conducted on the Bayesian information and linkage‐disequilibrium iteratively nested keyway (BLINK) in R Studio (v4.3.2). Manhattan and quantile‐quantile plots, created with the CMplot package in R, were used to identify significant SNPs across different chromosomes. To annotate genes associated with these significant SNPs in cowpea, the Phytozome database (https://phytozome.jgi.doe.gov) and the cowpea reference genome annotation *Vigna unguiculata* v1.2 were utilized.

### 2.4 Data Analysis

Statistical analyses were carried out using SAS 9.4 (SAS Institute Inc. 2019). Descriptive statistics for root phenotypes, averaged by genotype, were generated using PROC MEANS. Analysis of variance (ANOVA) for genotype and phenotype–genotype interactions was performed with PROC GLM. Correlation analysis was executed using R. Principal component analysis (PCA) was conducted in Python to determine the principal components accounting for the variance in root traits. Furthermore, Python was used to visualize the frequency distribution of all identified root traits.

## 3 Results

### 3.1 Root Phenotypic Assessment

**FIGURE 2:**
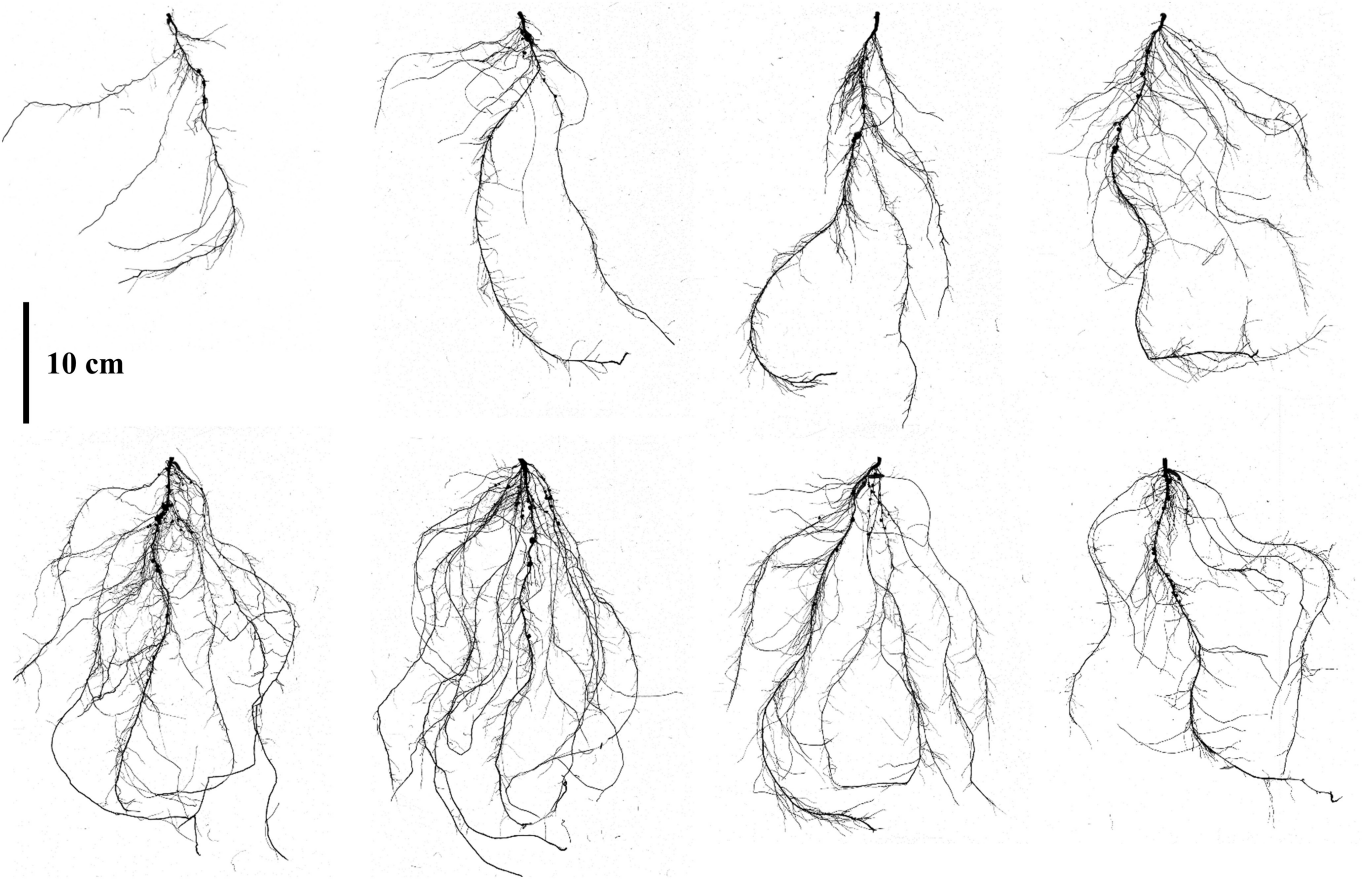
Variation among cowpea root genotypes analyzed during the present study. The scale bar represents 10 cm and applies uniformly to all root images, which were consistently resized to ensure a standard size.

The 222 cowpea accessions were evaluated for three root traits at the vegetative stage, with the roots harvested from PVC columns. ANOVA results indicated significant variations in some traits among the diverse cowpea lines, as presented in Table 1 and Figure 2. Root images were scanned using a width of 30 cm and a length of 40 cm transparent tray; a 10 cm scale bar was applied uniformly across all images, ensuring accurate measurements that correspond precisely to real dimensions and support reliable analysis (Figure 2). Among these findings, certain genotypes displayed exceptional performance in various agronomic traits. Specifically, genotype K261433 excelled across multiple traits, including SA, TN, and FN, suggesting a well‐developed root system. Genotype K002140 also showed strong performance in SA and TN, indicating its suitability for traits related to root structure and plant stability.

**TABLE 1:** Analysis variance (ANOVA) for all root traits.

Traits	Source	DF	Type III sum of square	Mean square	*f*	Pr > F
TRL	Accession	221	25,978,494	117,550	4.9391	< 0.0001
	Replication	3	139,403	46,468	1.9524	0.1199
SA	Accession	221	484,668	2193.07	5.5275	< 0.0001
	Replication	3	2040	679.94	1.7137	0.1629
AD	Accession	221	1.61148	0.007292	26.5997	< 0.0001
	Replication	3	0.00197	0.000656	2.3913	0.06827
RV	Accession	221	75.001	0.33937	13.4447	< 0.0001
	Replication	3	0.069	0.02307	0.9139	0.4338
TN	Accession	221	75,088,311	339,766	9.4742	< 0.0001
	Replication	3	146,307	48,769	1.3599	0.2541
FN	Accession	221	201895609.7	914,436	21.2014	< 0.0001
	Replication	3	306,454	102,151	2.3684	0.07001
PRL	Accession	221	90386.1	408.9869	2.82	< 0.0001
	Replication	3	975.2986	325.0995	2.24	0.0824
LRL	Accession	221	1,372,434	6210.109	8.76	< 0.0001
	Replication	3	4498.739	1499.58	2.12	0.0974

Abbreviations: AD, average diameter; FN, fork number; LRL, lateral root length; PRL, primary root length; RV, root volume; SA, surface area; TRL, total root length.

### 3.2 Correlation and Principal Component Analysis (PCA)

**FIGURE 3:**
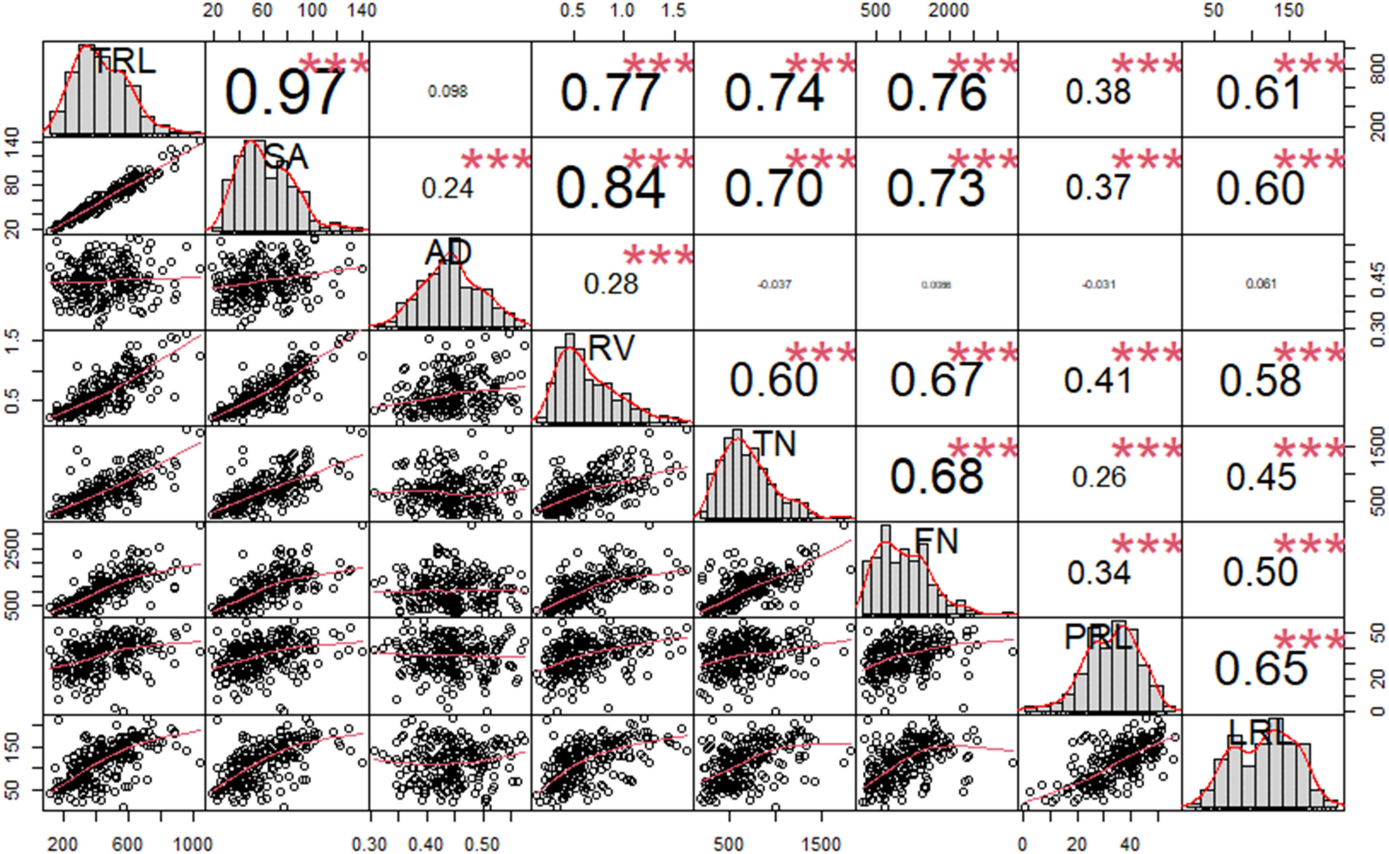
Correlation of root traits among different cowpea genotypes, ***p < 0.001 shows significance.

The estimation of the root traits revealed a significant positive correlation. Specifically, TRL revealed a strong positive correlation with SA (*r* = 0.97), RV (*r* = 0.77), TN (*r* = 0.74), FN (*r* = 0.76), PRL (*r =* 0.38), and LRL (*r* = 0.61) at *p <* 0.001. In addition, SA showed a significant positive correlation with all the traits AD (*r* = 0.24), RV (*r* = 0.84), TN (*r* = 0.70), FN (*r* = 0.73), PRL (*r* = 0.37), and LRL (*r* = 0.60) at *p* < 0.001. Similarly, RV was significantly positively correlated with TN (*r* = 0.60), FN (*r* = 0.67), PRL (*r* = 0.41), and LRL (*r* = 0.58) at *p <* 0.001. A correlation of TN, FN, PRL, and LRL showed a positive correlation with each other (Figure 3). The frequency distribution of the average root phenotypes (TRL, SA, AD, RV, TN, FN, PRL, and LRL) is further illustrated in Figure S1.

The descriptive statistics for the eight traits of TRL, SA, AD, RV, TN, FN, PRL, and LRL revealed notable variations among the traits. FN exhibited the greatest variability, with a wide range (201.67 to 3297) and the highest coefficient of variation (CV = 50.73%), indicating substantial diversity within the population. Conversely, AD displayed the least variability. Positive skewness was observed for several traits, including FN, which suggested the presence of outliers with higher values. In contrast, PRL and LRL showed negative skewness accompanied by kurtosis, indicating a distribution with a flatter peak and more evenly distributed data values, as shown in Table 2.

**TABLE 2:** Descriptive statistics of the root traits of all accessions.

Traits	Mean	SD	Min	Max	CV (%)	Skewness	Kurtosis
TRL	443.26	174.86	115	1037.19	39.45	0.57	0.08
SA	59.59	23.83	18.19	140.72	39.99	0.63	0.17
AD	0.44	0.05	0.31	0.57	11.88	0.11	−0.33
RV	0.63	0.30	0.14	1.62	47.98	0.92	0.41
TN	672.22	309.50	164	1820.33	46.04	0.86	0.69
FN	1074.32	544.99	201.67	3297	50.73	0.75	0.60
PRL	34.83	14.59	0.43	71.12	41.90	−0.16	−0.65
LRL	112.24	49.25	2.80	279.50	43.88	0.24	−0.49

Abbreviations: AD, average diameter; CV, coefficient of variation; FN, fork number; LRL, lateral root length; max, maximum; min, minimum; PRL, primary root length; RV, root volume; SA, surface area; SD, standard deviation; TRL, total root length.

**FIGURE 4:**
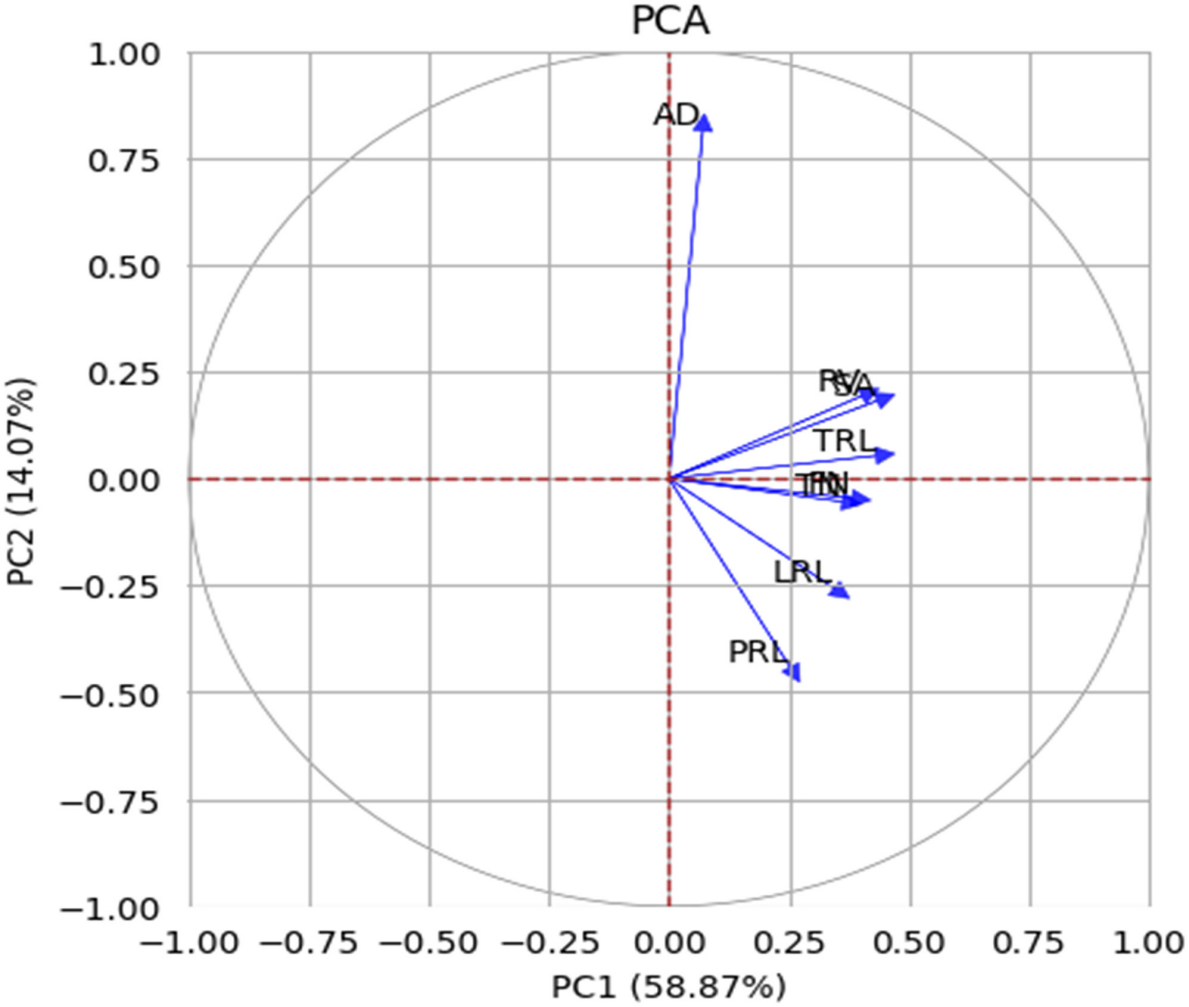
PCA Biplot of Root Traits in Diverse Cowpea Accessions. The biplot shows the variation in root traits among the cowpea accessions, with PC1 and PC2 accounting for 58.87% and 14.07% of the total variance, respectively.

The Principal Component Analysis (PCA) was conducted on the eight root traits, with most of the variability captured by the first two components. These components together explained a cumulative variance of 72.94%, with PC1 contributing 58.87% and PC2 accounting for 14.07% (Figure 4).

### 3.3 Marker‐Trait Associations

**FIGURE 5:**
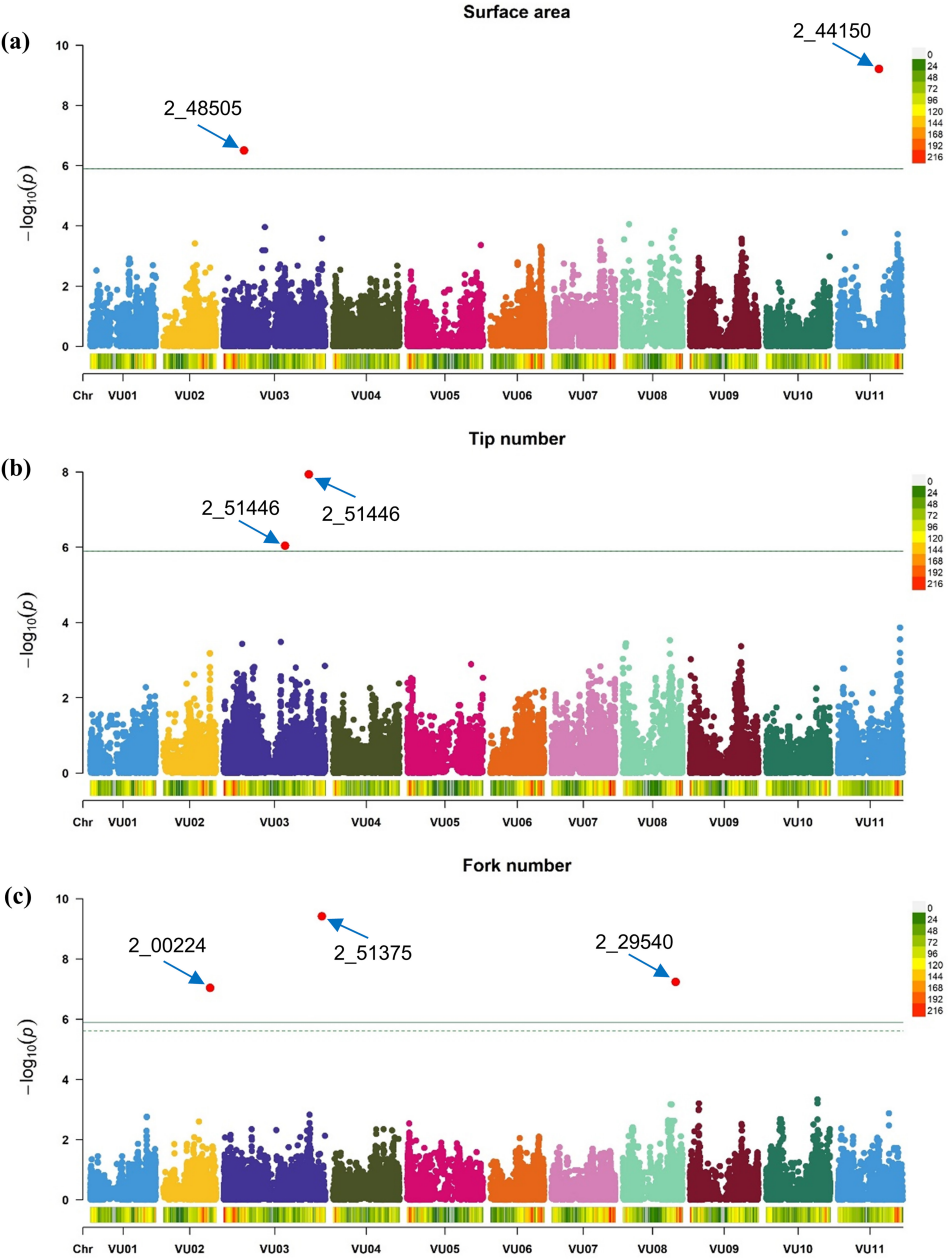
Manhattan plots of (a) Surface area, (b) Tip number, (c) Fork number. Each Manhattan plot is highlighted with red dots pointed out by blue arrows and labelled with marker names representing the significant SNPs identified by GWAS.

In this study, GWAS was conducted to investigate root traits using Bayesian Information and Linkage‐Disequilibrium Iteratively Nested Keyway (BLINK). The BLINK model successfully identified SNPs associated with the root traits. Among eight analyzed root traits, significant SNPs were identified for three key traits such as surface area (SA), tip number (TN), and fork number (FN). A total of seven significant SNPs associated with these traits are detailed in Table 3 and visualized in Figure 5.

**TABLE 3:** The significant single nucleotide polymorphism of the identified root traits.

Traits	SNP	Chromosome	Position	*p‐*value	MAF	−log10 (*p*)	Effect
SA	2_48505	VU03	13,163,420	3.17 × 10^−7^	0.36	6.50	−8.41
	2_44150	VU11	26,348,956	6.18 × 10^−10^	0.18	9.21	−12.17
TN	2_51446	VU03	39,402,755	9.13 × 10^−7^	0.07	6.04	−177.50
	2_11370	VU03	54,523,998	1.17 × 10^−8^	0.16	7.93	−183.12
FN	2_00224	VU02	30,035,803	9.23 × 10^−8^	0.24	7.03	−198.99
	2_51375	VU03	62,915,673	3.81 × 10^−10^	0.08	9.42	−331.51
	2_29540	VU08	33,896,575	5.85 × 10^−8^	0.11	7.23	270.70

Abbreviations: FN, Fork number; SA, Surface area; TN, Tip number.

**FIGURE 6:**
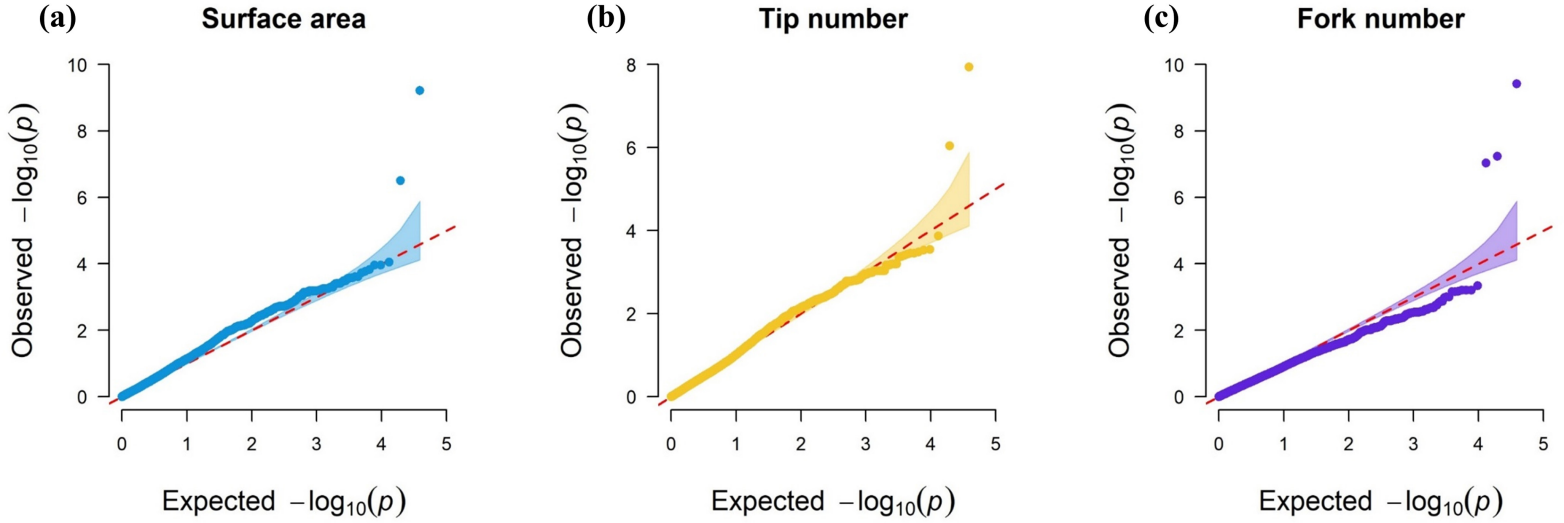
Quantile‐quantile (QQ) plots of (a) Surface Area, (b) Tip number, and (c) Fork number, traits falling above the threshold linear line showing significant marker‐trait associations.

Two significant SNPs were identified for SA, one on chromosome 3 (position 2_48505) and another on chromosome 11 (position 2_44150). Similarly, two significant SNPs associated with TN were found on chromosome 3, located at positions 2_51446 and 2_11370. For FN, three significant loci were identified: one on chromosome 2 (position 2_00224), another on chromosome 3 (position 2_51375), and a third on chromosome 8 (position 2_29540). Conversely, no significant SNPs were detected for the other five traits, such as TRL, AD, RV, PRL, and LRL illustrated in Figure S2. The Quantile‐quantile (QQ) plots for the aforementioned traits reveal significant marker‐trait associations. These associations are visually represented by data points that exceed the threshold linear line on the plot (Figure 6). These SNPs are crucial as they are associated with important root traits, offering valuable insights for the genetic improvement of cowpea.

### 3.4 Analysis of Candidate Genes

**FIGURE 7:**
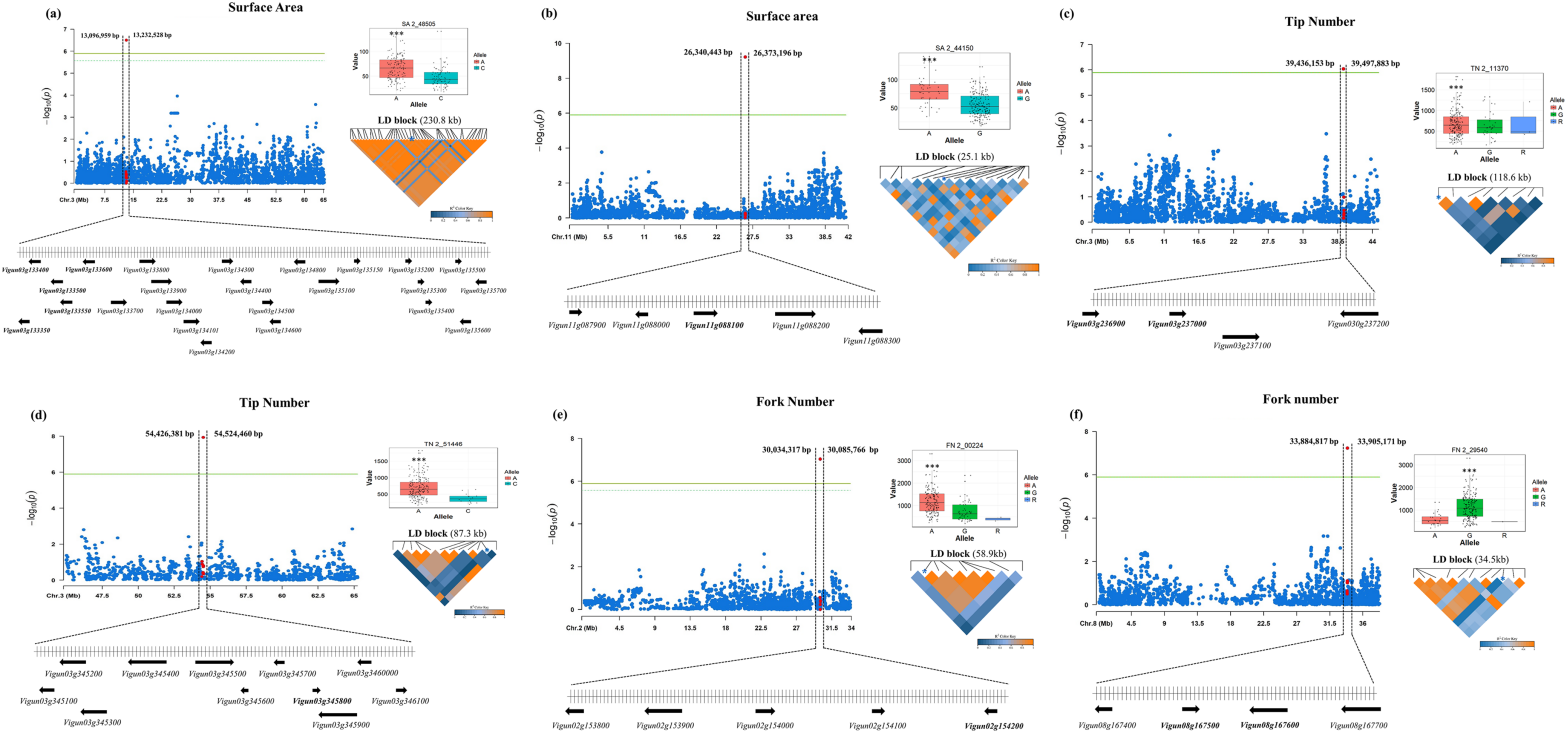
The results of the GWAS for three cowpea root traits: (a) SA‐VU03, (b) SA‐VU11, (c) TN (position 2_11370), (d) TN (position 2_51446), (e) FN‐VU02, and (f) FN‐VU08. Each trait is depicted with a Manhattan plot, where significant loci are highlighted as red dots above the green threshold lines. The genes that directly influence the root traits are indicated in bold. The box plots illustrate that different alleles at the significant SNP loci have distinct effects on these traits. The LD blocks demonstrate that the significant SNPs are within haplotype blocks. Moreover, the LD blocks show coefficients ranging from 0 to 1, with a color gradient from blue to orange representing the R^2^ values.

The analysis of genome‐wide linkage disequilibrium (LD) decay revealed an average *r* ^2^ value of 0.24 with a maximum of 0.47. Pairwise correlations between SNPs and the physical distances across the genome were plotted, estimating an average LD decay length of 416 kb (Lay et al. 2024). Subsequently, the concentration on shorter distances within haplotype blocks (linkage disequilibrium block) was identified using Haploview software, following the confidence interval criteria established by Gabriel et al. (2002). SNP LD heatmaps were generated using Bioinformatics tools (https://www.bioinformatics.com). This focused approach enabled the identification of 55 potential candidate genes associated with the three morphological root traits studied, as summarized in Table S2. Candidate genes were pinpointed by examining gene models within the LD block regions surrounding each significant SNP position using the cowpea reference genome database available on Phytozome (https://phytozome.jgi.doe.gov). Rather than conducting a broad and exhaustive search across the entire genome, which could be overly complex and yield too many results, the analysis focused on identifying 20 genes directly or indirectly linked to the root traits in question. These genes were associated with five out of the seven significant SNPs. The remaining identified genes were involved in various cellular components and contributed to a wide array of biological and molecular functions, as detailed in Table 4. This selective approach allowed us to focus on the most relevant genes for further study while still acknowledging the broader functional diversity of the other identified genes. To gain more detailed insights into gene functions, the Manhattan plots, box plots, and LD blocks generated narrowed down the genes located near significant loci for each identified trait. The regions above the threshold line in Figure 7 highlighted the SNPs that were significantly associated with these traits, marking candidate regions for further investigation. Notably, a significant SNP related to SA was identified on chromosome 3. This SNP was within an LD block spanning a distance of 230.8 kb, containing 24 genes (Figure 7a). Among these, five genes were predicted to have a direct function on SA. The gene *Vigun03g133350* was annotated as Phosphoenolpyruvate carboxylase/Phosphoenolpyruvic carboxylase, an enzyme involved in carbon fixation and plant metabolism, suggesting a role in root development. The other four genes, *Vigun03g133400*, *Vigun03g133500*, *Vigun03g133550*, and *Vigun03g133600*, were annotated as Brassinosteroid Insensitive 1‐Associated Receptor Kinase 1 (BAK1), a key regulator in brassinosteroid signaling crucial for plant growth and development, including root architecture. Additionally, six genes were predicted to have an indirect role on SA, including genes *Vigun03g134000*, *Vigun03g134101*, *Vigun03g134500*, *Vigun03g135400*, *Vigun03g135500*, and *Vigun03g135700*. These genes were annotated as 15‐cis‐phytoene desaturase/Plant‐type phytoene desaturase, Dynamin/Dynamin‐Like Protein Arc5, Large subunit ribosomal protein L44e (RPL44), tRNA (guanine(10)‐N(2))‐methyltransferase, Heterogeneous Nuclear Ribonucleoprotein G (RBMX, HNRNPG), and Vacuolar Iron Transporter Homolog 2.1, respectively. Furthermore, a candidate gene, *Vigun11g088100*, was identified on chromosome 11 within a 25.1 kb LD block (Figure 7b). This gene encodes a protein of unknown function (DUF688), potentially involved in stress signal transduction pathways or the regulation of stress‐responsive genes.

**TABLE 4:** The identified putative candidate genes are associated with significant SNPs. The table includes only those genes whose predicted functions are directly or indirectly related to cowpea root traits.

Traits	Gene name	Start (bp)	End (bp)	Function	Chromosome	SNP‐QTL	Position	Role	References
Surface area	*Vigun03g133350*	13,067,524	13,068,202	Phosphoenolpyruvate carboxylase/Phosphoenolpyruvic carboxylase	VU03	2_48505	13,163,420	Involved in ammonium stress, nitrate accumulated in roots, provision of malate guard cell and root nodules, improved plant growth and stress tolerance, drought response, environmental response, and tolerance to abiotic or chemical stress	(Pasqualini et al. 2001; González et al. 2003; Fukayama et al. 2006; Hyskova et al. 2014; Arias‐Baldrich et al. 2017; Waseem and Ahmad 2019; Liu et al. 2021)
	*Vigun03g133400* *Vigun03g133500* *Vigun03g133550* *Vigun03g133600*	13,074,648 13,089,755 13,091,381 13,095,227	13,082,167 13,091,381 13,092,828 13,101,529	Brassinosteroid insensitive 1‐associated receptor kinase 1 (BAK1)	VU03			ABA‐induced root growth and seed germination, regulate cell division during root development via interplaying with phytohormones (Auxin)	(Du et al. 2012; Shang et al. 2022)
	*Vigun03g134000*	13,138,757	13,141,597	15‐cis‐phytoene desaturase/Plant‐type phytoene desaturase	VU03			Influenced by the taproot colour, and storage root development	(Flores‐Ortiz et al. 2020; Li et al. 2021)
	*Vigun03g134101*	13,142,776	13,149,271	Dynamin/Dynamin‐Like Protein Arc5	VU03			Cassava leaves and tuber root development	(Cao, Liu, et al. 2019)
	*Vigun03g134500*	13,181,045	13,182,690	Large subunit ribosomal protein L44e (RPL44)	VU03			Abiotic stress response includes salt, drought, and heavy metals	(Liu et al. 2014)
	*Vigun03g135400*	13,274,617	13,278,258	tRNA (guanine(10)‐N(2))‐methyltransferase	VU03			Negatively expressed in the plant growth by delaying flowering and reducing primary root length.	(Guo et al. 2019)
	*Vigun03g135500*	13,283,406	13,285,614	Heterogeneous nuclear ribonucleoprotein G (RBMX, HNRNPG)	VU03			Negatively affects plant growth and abiotic stress tolerance	(Wang et al. 2016)
	*Vigun03g135700*	13,293,065	13,293,944	Vacuolar iron transporter homolog 2.1	VU03			Enhancing iron tolerance of the *Brassica napus* ; involved in iron supply to the developing nodule and symbiotic Nitrogen Fixation (SNF).	(Zhu et al. 2016; Ram et al. 2021)
	*Vigun11g088100*	26,340,210	26,344,619	Protein of unknown function (DUF688)	VU11	2_44150	26,348,956	Involved in mitigating the salt stress of the soybean	(Zaynab et al. 2023; Han et al. 2024)
Tip number	*Vigun03g236900* *Vigun03g237000*	39,436,153 39,457,692	39,438,705 39,462,481	BURP domain (BURP)	VU03	2_51446	39,402,755	Played an important role in plant metabolism and development; strong expression in plant responses to both biotic and abiotic stress, including salt, cold, and desiccation stress in root	(Ding et al. 2009; Sun et al. 2019; Chitkara et al. 2022)
	*Vigun03g345800*	54,503,286	54,504,670	Tetraspanin family (Tetraspannin)	VU03	2_11370	54,523,998	Plays a crucial role in primary root growth and lateral root development and redundant roles in leaf and root growth through negative regulation of cell proliferation; important functions in plant development, reproduction, and stress responses; regulates the size of flower organs and impacts various developmental processes in rice growth and development as the results of the interaction between tetraspanin AAF and auxin/JA	(Wang, Muto, et al. 2015; Reimann et al. 2017; Chen et al. 2019; Qin et al. 2024)
Fork number	*Vigun02g153900*	30,042,778	30,052,096	Branched‐Chain‐Amino‐Acid Aminotransferase 3, Chloroplastic	VU02	2_00224	30,035,803	Maintained root uptake amino acid, and multiple plant tissues	(Lee et al. 2007; Maloney et al. 2010)
	*Vigun02g154000*	30,065,608	30,067,441	Homeobox‐Leucine Zipper Protein Hat9	VU02			Involved in the stele and epidermis of the plant's root	(Perotti et al. 2021)
	*Vigun02g154200*	30,083,802	30,085,766	Xyloglucan Endotransglucosylase/Hydrolase Protein 6‐Related	VU02			Involved in cell elongation of Arabidopsis and tobacco; root hair; involved in cell wall modification processes during the growth and development of roots; responded to specific biotic or abiotic stress	(Vissenberg et al. 2000, 2001, 2003, 2005; Liu et al. 2007; Cao, Lv, and Li 2019; Ishida and Yokoyama 2022)
	*Vigun08g167500*	33,893,004	33,895,695	Weak chloroplast movement under blue light (WEMBL)	VU08	2_29540	33,896,575	Played an important role as an auxin and hormonal influence on plant	(Banaś et al. 2012)
	*Vigun08g167600*	33,896,533	33,901,565	Indole‐3‐glycerol‐phosphate synthase/Indoleglycerol phosphate synthetase	VU08			Involved in the biosynthesis of defensive metabolites of the insect on the maize	(Richter et al. 2021)

These findings pinpointed specific candidate genes within the LD block that were closely associated with the significant SNPs, providing valuable targets for further functional validation and exploration of their roles in root traits. The study focused on two SNPs located on chromosome 3 that were associated with TN (Figure 7c,d). The genes *Vigun03g236900* and *Vigun03g237000* encode proteins containing a BURP domain, which is implicated in cell wall modification and stress responses, and could be relevant to root development. Another gene, *Vigun03g3458000*, was annotated as a member of the tetraspanin family. Tetraspanins are membrane proteins involved in cell signaling, adhesion, and development, which may influence root architecture.

For FN, three candidate genes on chromosome 2 were identified within a 58.9 kb LD block (Figure 7e). The gene *Vigun02g153900* encoded Branched‐Chain‐Amino‐Acid Aminotransferase 3, Chloroplastic, which facilitates amino acid uptake in roots and other tissues. *Vigun02g154000* encodes Homeobox‐Leucine Zipper Protein Hat9, involved in the plant root epidermis development, and *Vigun02g154200* encodes Xyloglucan Endotransglucosylase/Hydrolase Protein 6 (XTH6), critical for cell wall modification during plant growth and development. On chromosome 8, two genes, *Vigun08g167500* and *Vigun08g167600*, encode Weak chloroplast movement under blue light (WEMBL) and indole‐3‐glycerol‐phosphate synthase/indoleglycerol phosphate synthetase, respectively. These genes are involved in hormone interactions and the biosynthesis of defensive metabolites (Figure 7f).

The presence of these genes within the LD blocks suggests they may influence root traits, such as SA, TN, and FN, through cell wall properties, stress responses, or metabolic pathways. Furthermore, distinct allelic effects at significant SNP loci indicated that the identified genetic variants could have a functional impact on these traits. The LD blocks analysis provided additional context by showing the haplotype blocks containing causal variants or other candidate genes.

## 4 Discussion

Cowpea is a vital annual legume crop with global significance, particularly in Sub‐Saharan Africa, where it serves as a staple food for millions of people (Ngalamu et al. 2015; Enyiukwu et al. 2018; Kebede and Bekeko 2020). One of the key strengths of cowpea is its resilience, thriving under low soil fertility and dry conditions, making it highly suitable for cultivation in environments with limited inputs and water availability (Nkhoma et al. 2020). This resilience is largely attributed to its root architecture, which plays a crucial role in nutrient acquisition, environmental stress tolerance, soil health improvement, and overall sustainability of agricultural systems. These factors collectively influence the productivity and ecological benefits of cowpea. The present study integrates root phenotyping over two growing seasons in the same environment with yield trials conducted in those conditions. It focuses on understanding how specific root traits, such as root depth and primary root elongation, root surface area, root tip number, and root fork number, contribute to the performance of cowpea genotypes. Previous research has highlighted that root depth and primary root elongation are more reliable selection parameters for legumes than root length density and dry root matter (Hamblin and Tennant 1987). The current study finds significant variation in these traits among the cowpea genotypes examined, providing evidence for the genetic diversity in root architecture within the species. Earlier studies on cowpea root phenotyping, such as those by Adu et al. (2019), Ittah and Arua (2017), Comas et al. (2013), and Gwathmey et al. (1992), have similarly reported significant variation in root traits across different cowpea genotypes. These variations underscore the importance of understanding root architecture for selecting and breeding cowpea varieties that can better withstand environmental challenges, ultimately leading to improved yields and sustainability in diverse growing conditions.

**FIGURE 8:**
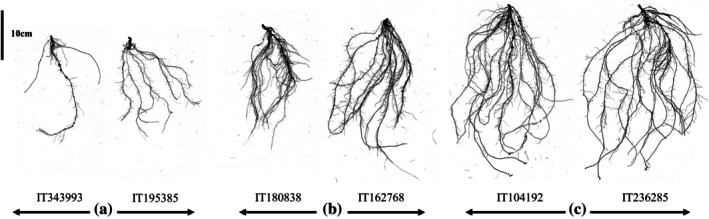
This figure illustrates the diversity in root system architecture among some selected cowpea genotypes, highlighting differences in SA, TN, and root depth. (A) IT343993 and IT195385 are examples of genotypes with low SA, low TN, and shallow root systems. (B) IT162768 and IT180838 are genotypes exhibiting high SA and high TN but with shallow root systems. (C) IT104192 and IT236285 are genotypes demonstrating high surface area, high density, high tip number, and deep root systems.

Cowpea genotypes exhibit significant variation in seedling root architecture, which has important agronomic implications. For instance, TRL shows an average of 443.26 cm across genotypes, with some exceeding 1039.17 cm. Similarly, SA averages 59.59 cm^2^, with certain genotypes surpassing 140.72 cm^2^. These differences illustrate the diversity in root system characteristics within the cowpea germplasm. Figure 8 provides a visual representation of this variation and showcases the range of root phenotypes from sparse and shallow (A) to dense and deep root systems (C). These variations in root architecture traits highlight the potential for targeted breeding to improve root system characteristics for enhanced resource acquisition and stress tolerance.

The effectiveness of root SA is particularly important for improving nutrient acquisition from the soil, a key factor in crop productivity. Root traits such as SA, TN, and FN collectively contribute to enhancing resource acquisition. Root tips facilitate soil exploration and nutrient uptake, while root forks improve root density and coverage, enhancing the plant's ability to access water and minerals in diverse soil conditions (J. Lynch 1995; Lynch and Brown 2012; Comas et al. 2013; Koevoets et al. 2016). Given the role of root SA in resource acquisition, breeding for specific root traits is a promising strategy for enhancing crop production, especially on limited arable land and under the pressures of a changing climate (Orman‐Ligeza et al. 2014). Root traits like SA are particularly valuable in improving plant productivity in conditions of climate change, such as drought, where robust root SA can significantly impact crop survival and yield. This study highlights significant variation in root SA, TN, and FN traits among cowpea genotypes, and these traits have been associated with improved root hair development, drought tolerance, mineral uptake, increased lateral root formation, general root growth, and development. Additionally, the root architecture of cowpea has been studied by Burridge et al. (2016), who found significant variation in basal root whorl number and basal root number under field conditions. Similarly, in the current study, significant differences have been observed in root traits such as branching, surface area, tip number, fork number, and primary root length among cowpea genotypes, measured at two time points within the same environment. These findings suggest that there is considerable genetic diversity in cowpea root architecture, which can be harnessed to develop varieties with improved performance under various environmental conditions.

Principal Component Analysis (PCA) in cowpea, as reported by Adu et al. (2019), identified several key traits that significantly contribute to variation in cowpea root architecture and growth. Among the top 50% of the most crucial traits were soil and root tissue angles, shoot and root diameter, root biomass, hypocotyl root length, root count, and lateral root density. These traits play a critical role in determining how cowpea plants interact with their environment, particularly in terms of nutrient and water acquisition, which are essential for growth and productivity. In a more recent study by Mohammed et al. (2022), PCA was also used to analyze cowpea root and seed traits, revealing important correlations between these traits. For instance, TRL showed a highly positive correlation with SA (*r* = 0.97), TN (*r* = 0.74), and FN (*r* = 0.76). This indicates that as the total root length increases, there is a corresponding increase in the surface area, tip number, and fork number, suggesting that these traits are closely related and collectively contribute to the plant's ability to explore soil resources effectively.

GWAS enable high‐resolution mapping to identify genes and SNPs linked to various traits in large populations (Mamo et al. 2014). In this study, GWAS was employed to address gaps in the genomic understanding of root traits in cowpea. Association between SNPs and eight root traits across diverse cowpea germplasm identified seven significant SNPs linked to three key root traits SA, TN, and FN. For SA, two significant SNPs were found on chromosome 3 (position 2_48505) and chromosome 11 (position 2_44150). For TN, two significant SNPs were located on chromosome 3 at positions 2_51446 and 2_11370. For FN, three significant SNPs were identified on chromosome 2 (position 2_00224), chromosome 3 (position 2_51375), and chromosome 8 (position 2_29540). These findings highlight crucial loci for breeding programs aimed at improving the root architecture of cowpea. Notably, chromosome 3 is associated with SA, with 11 genes playing direct and indirect roles in root traits (Table 4). Among these genes, the gene *Vigun03g133350* encodes for phosphoenolpyruvate carboxylase/phosphoenolpyruvic carboxylase, an enzyme central to carbon fixation in C4 and CAM plants (Pasqualini et al. 2001). This enzyme also contributes to organic acid biosynthesis, supports TCA cycle intermediates, and helps manage pH in rice (Fukayama et al. 2006), ion balance, and stress responses (González et al. 2003; Hyskova et al. 2014; Arias‐Baldrich et al. 2017; Waseem and Ahmad 2019; Liu et al. 2021). Additionally, genes such as *Vigun03g133400*, *Vigun03g133500*, *Vigun03g133550*, and *Vigun03g133600* are linked to Brassinosteroid insensitive 1‐associated receptor kinase 1 (BAK1), a key regulator of plant growth and development. BAK1 is part of the somatic embryogenesis receptor‐like kinase (SERK) subfamily, which is known to influence root development in Arabidopsis (Du et al. 2012; Planas‐Riverola et al. 2019). This study also identified QTLs that are closely linked to those previously reported for cowpea seed weight (Wu et al. 2024). Similarly, chromosome 3 regions linked to stem diameter traits were identified by Burridge et al. (2017). Such overlaps suggest that these root‐related loci may influence genetic regions critical for productivity, warranting further exploration. Moreover, on chromosome 11, the gene *Vigun11g088100* encodes a protein of unknown function (DUF688). This protein is believed to play a crucial role in plant growth and development, particularly under stressful environmental conditions (Zhao et al. 2021). In *Glycine max* , the *GmDUF688* protein has been shown to alleviate the effects of abiotic stresses, especially salt stress, and is expressed in roots, nodules, leaves, and flowers (Zaynab et al. 2023).

The genes *Vigun03g236900* and *Vigun03g237000*, associated with TN, encode proteins containing the BURP domain, plant‐specific and involved in various developmental processes and stress responses (Wang, Wu, et al. 2015). The BURP family plays a significant role in stress responses in legumes, with upregulation under abiotic stresses in roots (Chitkara et al. 2022). For instance, *OsBURP11* was found to be strongly expressed in roots (Ding et al. 2009), while nine GhBURPs in cotton exhibited dominant expression in specific organs, including roots, stems, stamens, and ovules (Sun et al. 2019). In *Arabidopsis thaliana* , the BURP genes *AtRD22* and *AtUSPL1* were identified as crucial for drought tolerance, with *AtUSP1* being prevalent in roots (Harshavardhan et al. 2014). Similarly, in *Medicago truncatula* , BURP genes exhibited varied expression in both aerial (stem, leaf, and flowers) and underground tissues (roots) in response to drought stress (Li et al. 2016). The gene *Vigun03g345800*, also associated with TN, encodes a member of the tetraspanin family, a group of evolutionarily conserved integral membrane proteins important for plant development, particularly in root tissues. Previous studies reported preferential expression of tetraspanins in rice root tissues (*OsTET5*, *OsTET8*, and *OsTET12*) and *Arabidopsis* (*TET13*), where they are involved in primary and lateral root development (Mani et al. 2015; Wang, Muto, et al. 2015; Reimann et al. 2017; Qin et al. 2024). Additionally, Auxin Activation Factor (AAF) orthologs in orchids, identified as part of the tetraspanin gene family, enhance drought tolerance and increase lateral root formation (Chen et al. 2019).

The gene *Vigun02g154200*, associated with FN, encodes xyloglucan endotransglucosylase/Hydrolase Protein 6‐related, an enzyme reported to be present in the roots of diverse vascular plants (Vissenberg et al. 2003). This enzyme enhances the initiation of root hair in Arabidopsis (Vissenberg et al. 2001), and its donor substrates in the elongation zone of *Arabidopsis* roots facilitate cell wall loosening and synthesis integration (Vissenberg et al. 2000). The genes *AtXTH17* and *AtXTH18* are expressed in all cell types within the elongation and differentiation regions of the root and are upregulated in response to auxin (Vissenberg et al. 2005). *AtXTH21* (*At2g18800*) is mainly expressed in roots and flowers (Liu et al. 2007). Furthermore, transgenic and mutant plant analyses have revealed that *AtXTH10* regulates cell wall structure, affecting root growth and development (Cao, Lv, and Li 2019). Additionally, phylogenetic analyses by Osato et al. (2006) revealed that the *Arabidopsis* XTH genes *AtXTH17*, *AtXTH18*, *AtXTH19*, and *AtXTH20* are closely related and predominantly expressed in roots.

## 5 Conclusion

Root traits play a pivotal role in enhancing plant productivity, especially under challenging environmental conditions such as drought. Understanding root‐soil interactions can lead to improvements in plant resilience and overall performance. Effective root phenotyping, which involves the precise measurement and comprehensive analysis of root characteristics, is essential for advancing plants' capacity to thrive in suboptimal environments. This study underscores the significance of root architecture in uncovering mechanisms to enhance productivity under adverse conditions, supporting the development of cultivars better suited to climate challenges. Through association mapping of 222 cowpea accessions, the research identified 55 genes associated with three principal root traits, with seven significant SNPs associated with SA, TN, and FN. The positive correlation observed between SA and other root traits suggests that enhancing SA could lead to concurrent improvements in various aspects of root development. This study provides a solid foundation for genetic improvements in breeding programs with the implementation of high‐throughput phenotyping at the vegetative growth stage, enabling efficient identification of genotypes with favorable root traits. This facilitates the selection of plants exhibiting enhanced root development, improved stress tolerance, and more effective mineral uptake. Ultimately, these advancements pave the way for the development of more resilient and productive cowpea cultivars.

## Author Contributions

L.L. and S.M. contributed to the preparation of original draft, conducted the research investigation, illustrated the figures and performed formal data analysis. W.K., M.S.I., and A.G. conducted the research investigation, reviewed and edited the draft. H.J., and Y.S.C. provided critical validation of the results and edited and reviewed the draft. Y.K. conceptualized and supervised the study, edited the draft and secured the fundings.

## Supporting information


Figure S1.

Figure S2.

**Table S1.** The list of countries of origin for the germplasm.

**Table S2.** The putative candidate genes are related directly and indirectly to four root traits.

## Data Availability

The data that support the findings of this study are available from the corresponding author upon reasonable request.
