# Supplementary figures and images for: Advanced High‐Throughput Root Phenotyping and GWAS Identifies Key Genomic Regions in Cowpea During Vegetative Growth Stage
Source: Physiol Plant. 2025 Jul 3;177(4):e70375. doi: 10.1111/ppl.70375 (PMC12232088; doi:10.1111/ppl.70375)

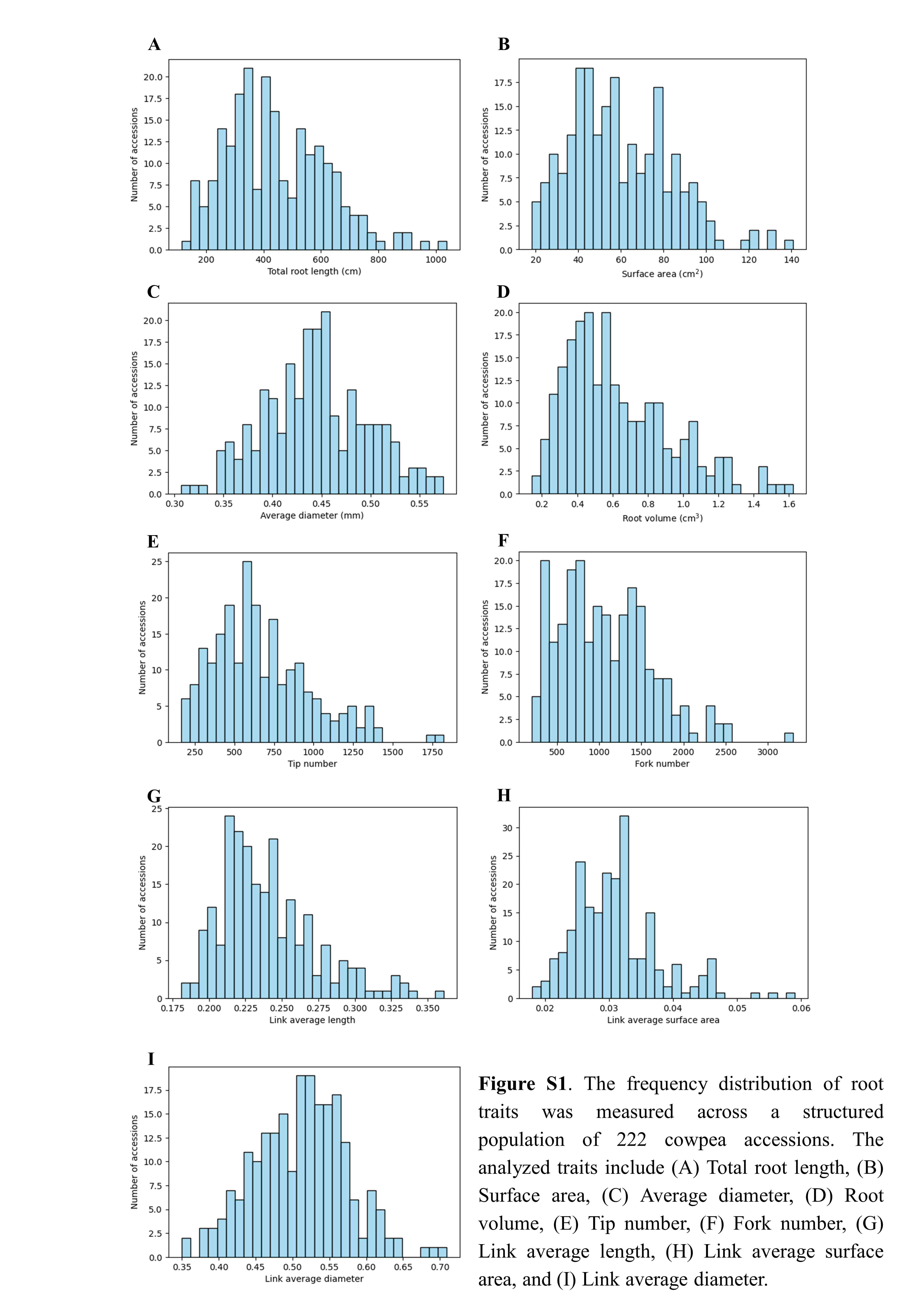

Supplement: Supplementary file 1 — Figure S1. [file PPL-177-e70375-s004.tif]

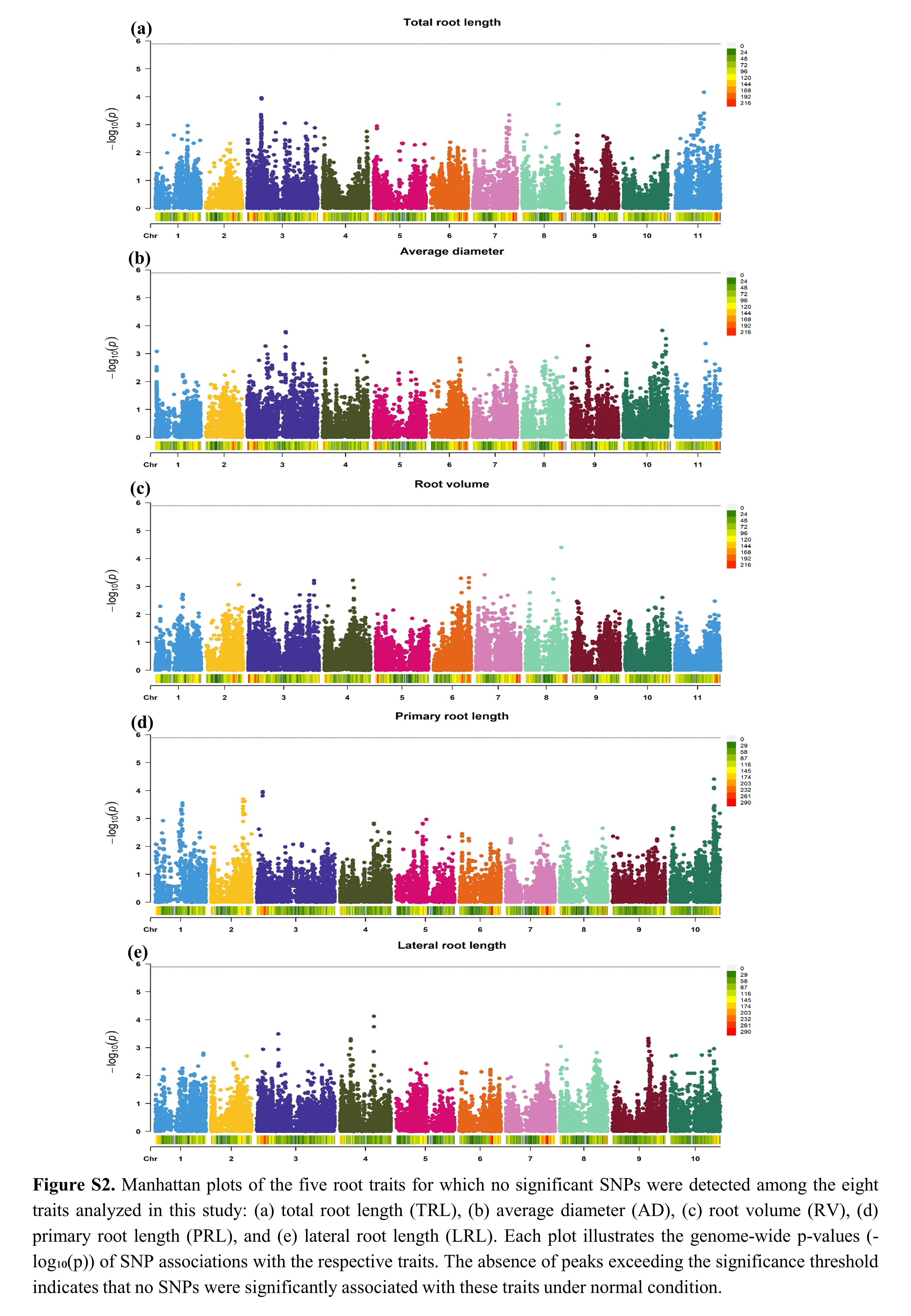

Supplement: Supplementary file 2 — Figure S2. [file PPL-177-e70375-s001.tif]
